# Supplementary material for: Computer-vision object tracking for monitoring bottlenose dolphin habitat use and kinematics
Source: PLoS One. 2022 Feb 3;17(2):e0254323. doi: 10.1371/journal.pone.0254323 (PMC8812882; doi:10.1371/journal.pone.0254323)
Supplement: S1 Table — (PDF) [file pone.0254323.s001.pdf]

Computer-vision object tracking for monitoring bottlenose dolphin habitat use and kinematics - Supporting information

1 Supporting information - Results

Table S1. Kolmogorov-Smirnov Session Comparison

	Blk.		Speed Δ_{ks}	Speed α	Yaw Δ_{ks}	Yaw α	Yaw Rate Δ_{ks}	Yaw Rate α
OTS	1	2	0.187	< 0.001	0.028	< 0.001	0.047	< 0.001
	1	3	0.095	< 0.001	0.021	0.025	0.034	< 0.001
	1	4	0.080	< 0.001	0.019	0.049	0.057	< 0.001
	1	5	0.079	< 0.001	0.021	0.027	0.035	< 0.001
	2	3	0.096	< 0.001	0.028	< 0.001	0.017	0.099
	2	4	0.111	< 0.001	0.025	0.003	0.028	< 0.001
	2	5	0.110	< 0.001	0.023	0.012	0.016	0.148
	3	4	0.026	0.002	0.019	0.046	0.025	0.004
	3	5	0.026	0.003	0.022	0.012	0.010	0.685
	4	5	0.018	0.093	0.013	0.403	0.030	< 0.001
ITS	1	2	0.059	< 0.001	0.028	< 0.001	0.022	0.017
	1	3	0.021	0.019	0.020	0.039	0.008	0.871
	1	4	0.059	< 0.001	0.028	0.001	0.021	0.020
	2	3	0.061	< 0.001	0.023	0.009	0.028	< 0.001
	2	4	0.043	< 0.001	0.010	0.638	0.008	0.940
	3	4	0.068	< 0.001	0.029	< 0.001	0.028	< 0.001

	Blk.		Speed σ Δ_{ks}	Speed σ α	Yaw σ Δ_{ks}	Yaw σ α	Yaw Rate σ Δ_{ks}	Yaw Rate σ α
OTS	1	2	0.047	< 0.001	0.035	< 0.001	0.076	< 0.001
	1	3	0.012	0.434	0.026	0.002	0.053	< 0.001
	1	4	0.025	0.004	0.029	< 0.001	0.062	< 0.001
	1	5	0.014	0.249	0.015	0.222	0.040	< 0.001
	2	3	0.047	< 0.001	0.031	< 0.001	0.033	< 0.001
	2	4	0.065	< 0.001	0.039	< 0.001	0.043	< 0.001
	2	5	0.051	< 0.001	0.048	< 0.001	0.043	< 0.001
	3	4	0.025	0.005	0.016	0.153	0.014	0.264
	3	5	0.008	0.889	0.026	0.002	0.026	0.002
	4	5	0.025	0.003	0.032	< 0.001	0.035	< 0.001
ITS	1	2	0.033	< 0.001	0.108	< 0.001	0.092	< 0.001
	1	3	0.027	0.001	0.012	0.423	0.016	0.139
	1	4	0.040	< 0.001	0.096	< 0.001	0.086	< 0.001
	2	3	0.046	< 0.001	0.103	< 0.001	0.100	< 0.001
	2	4	0.014	0.303	0.014	0.264	0.026	0.003
	3	4	0.056	< 0.001	0.093	< 0.001	0.095	< 0.001
